# Measuring Engagement in Provider-Guided Digital Health Interventions With a Conceptual and Analytical Framework Using Nurse WRITE as an Exemplar: Exploratory Study With an Iterative Approach

**DOI:** 10.2196/57529

**Published:** 2024-07-22

**Authors:** Yan Wang, Annette DeVito Dabbs, Teresa Hagan Thomas, Grace Campbell, Heidi Donovan

**Affiliations:** 1 Department of Health & Community Systems, School of Nursing University of Pittsburgh Pittsburgh, PA United States; 2 Department of Acute & Tertiary Care, School of Nursing University of Pittsburgh Pittsburgh, PA United States; 3 Department of Health Promotion & Development, School of Nursing University of Pittsburgh Pittsburgh, PA United States; 4 School of Nursing Duquesne University Pittsburgh, PA United States; 5 Department of Gynecology, Oncology, and Reproductive Sciences, School of Medicine University of Pittsburgh Pittsburgh, PA United States

**Keywords:** engagement, digital health intervention, framework, symptom management, eHealth, gynecological cancer

## Abstract

**Background:**

Limited guidance exists for analyzing participant engagement in provider-guided digital health interventions (DHIs). System usage is commonly assessed, with acknowledged limitations in measuring socio-affective and cognitive aspects of engagement. Nurse WRITE, an 8-week web-based nurse-guided DHI for managing symptoms among women with recurrent ovarian cancer, offers an opportunity to develop a framework for assessing multidimensional engagement.

**Objective:**

This study aims to develop a conceptual and analytic framework to measure socio-affective, cognitive, and behavioral engagement with provider-guided DHIs. We then illustrate the framework’s ability to describe and categorize engagement using Nurse WRITE as an example.

**Methods:**

A sample of 68 participants from Nurse WRITE who posted on the message boards were included. We adapted a prior framework for conceptualizing and operationalizing engagement across 3 dimensions and finalized a set of 6 distinct measures. Using patients' posts, we created 2 socio-affective engagement measures—total count of socio-affective engagement classes (eg, sharing personal experience) and total word count—and 2 cognitive engagement measures—total count of cognitive engagement classes (eg, asking information-seeking questions) and average question completion percentage. Additionally, we devised behavioral engagement measures using website data—the total count of symptom care plans and plan reviews. k-Means clustering categorized the participants into distinct groups based on levels of engagement across 3 dimensions. Descriptive statistics and narratives were used to describe engagement in 3 dimensions.

**Results:**

On average, participants displayed socio-affective engagement 34.7 times, writing 14,851 words. They showed cognitive engagement 19.4 times, with an average of 78.3% completion of nurses' inquiries. Participants also submitted an average of 1.6 symptom care plans and 0.7 plan reviews. Participants were clustered into high (n=13), moderate (n=17), and low engagers (n=38) based on the 6 measures. High engagers wrote a median of 36,956 (IQR 26,199-46,265) words. They demonstrated socio-affective engagement approximately 81 times and cognitive engagement around 46 times, approximately 6 times that of the low engagers and twice that of the moderate engagers. High engagers had a median of 91.7% (IQR 82.2%-93.7%) completion of the nurses’ queries, whereas moderate engagers had 86.4% (IQR 80%-96.4%), and low engagers had 68.3% (IQR 60.1%-79.6%). High engagers completed a median of 3 symptom care plans and 2 reviews, while moderate engagers completed 2 plans and 1 review. Low engagers completed a median of 1 plan with no reviews.

**Conclusions:**

This study developed and reported an engagement framework to guide behavioral intervention scientists in understanding and analyzing participants’ engagement with provider-guided DHIs. Significant variations in engagement levels across 3 dimensions highlight the importance of measuring engagement with provider-guided DHIs in socio-affective, cognitive, and behavioral dimensions. Future studies should validate the framework with other DHIs, explore the influence of patient and provider factors on engagement, and investigate how engagement influences intervention efficacy.

## Introduction

Active participant engagement is pivotal to maximizing intervention benefits and enhancing health outcomes [1]. Therefore, measuring and evaluating engagement levels is essential for drawing valid conclusions about intervention efficacy. In recent decades, digital technologies, including mHealth and eHealth, have been extensively integrated into interventions targeting symptom management [2,3] and behavior change promotion [4-6]. Studies indicate that cancer survivors’ use of technology and engagement with digital health interventions (DHIs) tends to decline over time [7,8], possibly due to participant intervention fatigue, cognitive overload, habituation to frequent contact, and negative emotions [9]. While human support is often considered a potential remedy for this issue in provider-guided DHIs [10], previous research has not adequately accounted for the complexity of patient engagement with provider-guided DHIs.

Although there is no consensus on the definition of engagement, it is predominantly seen as a multidimensional construct [11], including socio-affective, cognitive, and behavioral dimensions. Extant literature heavily relies on assessing behavioral engagement through system usage patterns, such as website visits, app usage, or posting frequency [12-20], which has been criticized in recent systematic reviews [21]. Research indicates that solely measuring system usage does not guarantee that participants are receiving the intended dosage (the theoretically or empirically determined amount of exposure) of an intervention or that their interactions with the intervention are meaningful and effective. [22,23]. Attempts have been made to gauge participants’ cognitive and affective engagement through subjective measures such as self-report questionnaires [24], ecological momentary assessments [25], and qualitative methods like interviews and think-aloud activities conducted during or after the intervention [26]. However, these measures have notable limitations. Exit interviews, for instance, fail to capture participants' real-time experiences during the intervention and may be compromised by recall bias. This issue was highlighted in a previous study on cancer survivors’ engagement with an app, where participants struggled to recall app functions or usage details [26]. Complementing this line of research, many researchers emphasize the importance of using multiple methods and integrating diverse data sets that encompass cognitive and socio-affective engagement alongside usage data to unravel the complexities inherent in the concept and measurement of engagement [21,27]. Building on these studies, we sought to develop a comprehensive conceptual and analytic framework to describe and assess patient engagement with a provider-guided DHI based on a more conceptual understanding of dimensions of engagement, including socio-affectivity [28], cognition [28], and behavior.

The WRITE Symptoms Study (NRG Oncology’s GOG-259; NR010735) was a 3-arm web-based symptom management intervention designed for women with recurrent cancer [29]. The Nurse WRITE intervention arm was a nurse-guided DHI conducted on web-based asynchronous message boards. A study nurse guided participants using the representational approach to patient education [30,31]. This disease-agnostic approach thoroughly assesses participants' symptom beliefs before offering personalized symptom management recommendations and individualized problem-solving support. The other intervention arm adopted a similar process, replacing nurse interaction with self-directed web-based modules for participants. Both Nurse and self-directed WRITE demonstrated efficacy in improving symptom control, as measured by the Symptom Representation Questionnaire [31], compared to the control arm. However, there was a notable high variation in patient completion of the intervention. Importantly, all interactions between the nurses and participants are documented verbatim on the message board, providing an excellent opportunity to examine the complexity of the engagement.

In a previous study, we constructed the “MedNgage Dataset” [28] that covers coded cognitive and socio-affective engagement from the library of nurse-participant posts on asynchronous message boards in Nurse WRITE. Drawing from the social presence model [32], the cognitive science of grounding in communication [33], and linguistic discourse theories [34], we collaborated with linguists and used iterative coding to develop the socio-affective and cognitive engagement (SACe) conceptual framework to capture these dimensions of engagement. First, we specifically characterized socio-affective engagement in provider-guided DHIs as the participants' endeavors to establish emotional connections with providers during activities like communication and collaboration. Second, cognitive engagement is defined as the participant’s coordination of intervention content and process with providers (eg, the patient understands the content provided and effectively follows the intervention activities, such as answering protocolized questions). In a complex provider-guided DHI like Nurse WRITE, building emotional connections (socio-affective engagement) and participants’ understanding of the intervention (cognitive engagement) can empower patients to explore and adopt the strategies best suited to managing their symptoms. This is the third dimension, behavioral engagement, and by taking action, participants' comprehension of skills and processes is enhanced, further reinforcing their connection with the provider.

Expanding on this foundation, the current study has 2 primary objectives. First, it aims to finalize a conceptual and analytic framework, building on the SACe, that quantifies engagement levels with provider-guided DHIs across 3 dimensions: socio-affective, cognitive, and behavioral. Second, it applies the framework to measure and categorize patients' engagement patterns with the Nurse Write intervention as an exemplar. This provides guidance and insights for future evaluations of engagement in provider-guided DHIs.

## Methods

### Overview

This study was an ancillary analysis of 68 patients with recurrent ovarian cancer from the Nurse WRITE arm of the WRITE Symptoms Study. The study included qualitative data from patients’ asynchronous message boards and quantitative website tracking data.

### Ethical Considerations

The WRITE symptoms interventions were approved by the University of Pittsburgh Human Research Protection Office Institutional Review Board and each of the 34 participating clinical sites (number PRO09090033), allowing for deidentified data sharing. Informed consent was obtained from all participants, and they were compensated for their participation [29]. All activities and questionnaires were conducted using a secure, password-protected website developed at the University of Pittsburgh, ensuring accurate and Health Insurance Portability and Accountability Act-compliant data collection. This secondary data analysis used the deidentified data set, posing no risks for participants.

### Setting and Sample

The parent trial enrolled 497 women with recurrent ovarian cancer from various Gynecologic Oncology Group (GOG) and NRG Oncology-affiliated sites [29]. To be eligible for the parent study, participants had to be 18 years or older, diagnosed with recurrent or persistent ovarian, fallopian, or primary peritoneal cancer, and possess a GOG performance status of less than 3, indicating that they are ambulatory and capable of self-care but unable to perform work activities, with more than 50% of waking hours spent up and about [35,36]. Additionally, they were required to experience at least 3 symptoms associated with cancer or its treatment (eg, pain, fatigue, and neuropathy) and be able to read and write in English. Details of the study design, protocol for Nurse WRITE (intervention group), and results have been described elsewhere [29]. Among the 166 participants randomized to Nurse WRITE, 141 women met the study criteria and posted at least once on the message board. For this study, we selected a convenience sample of the first 68 participants randomized to Nurse WRITE, representing 50% of the total number of message board posts from the pool of 141 participants. This selection aimed to ensure alignment with the sample in the previous “MedNgage” study [28] and to manage the qualitative analysis workload.

### Asynchronous Message Boards

Nurse WRITE was delivered by nurses highly trained to the protocol that delivered the intervention with high fidelity, offering information and support tailored to each participant's needs [37]. Through open-ended prompts and protocolized questions, participants engaged in dynamic interactions with nurse interventionists on a private asynchronous message board on the study website, including expressing symptom perceptions, reflecting, discussing coping efforts, and collaborating with nurses in developing personalized symptom care plans for 3 participant-identified target symptoms during the 8-week intervention period. To create a symptom care plan, participants needed to go through 6 out of 7 key intervention elements (representational assessment; identifying and exploring gaps, errors, and confusions; creating conditions for conceptual change; introducing replacement information; summary; goal setting and planning). The seventh element is reviewing and revising the symptom care plan after 2 weeks. This involved assessing goal achievement, strategy implementation, and any difficulties encountered. Participants were then instructed to try the revised plan and review the results following the same approach as needed on their own.

### Data Collection and Analysis

#### Participant Data

Patient demographic characteristics were assessed with the Center for Research in Chronic Disorders Socio-Demographic Survey [38] for age, education, race, marital status, and employment.

#### Aim 1: Finalize a Conceptual and Analytic Framework With Engagement Measures

**Figure 1:**
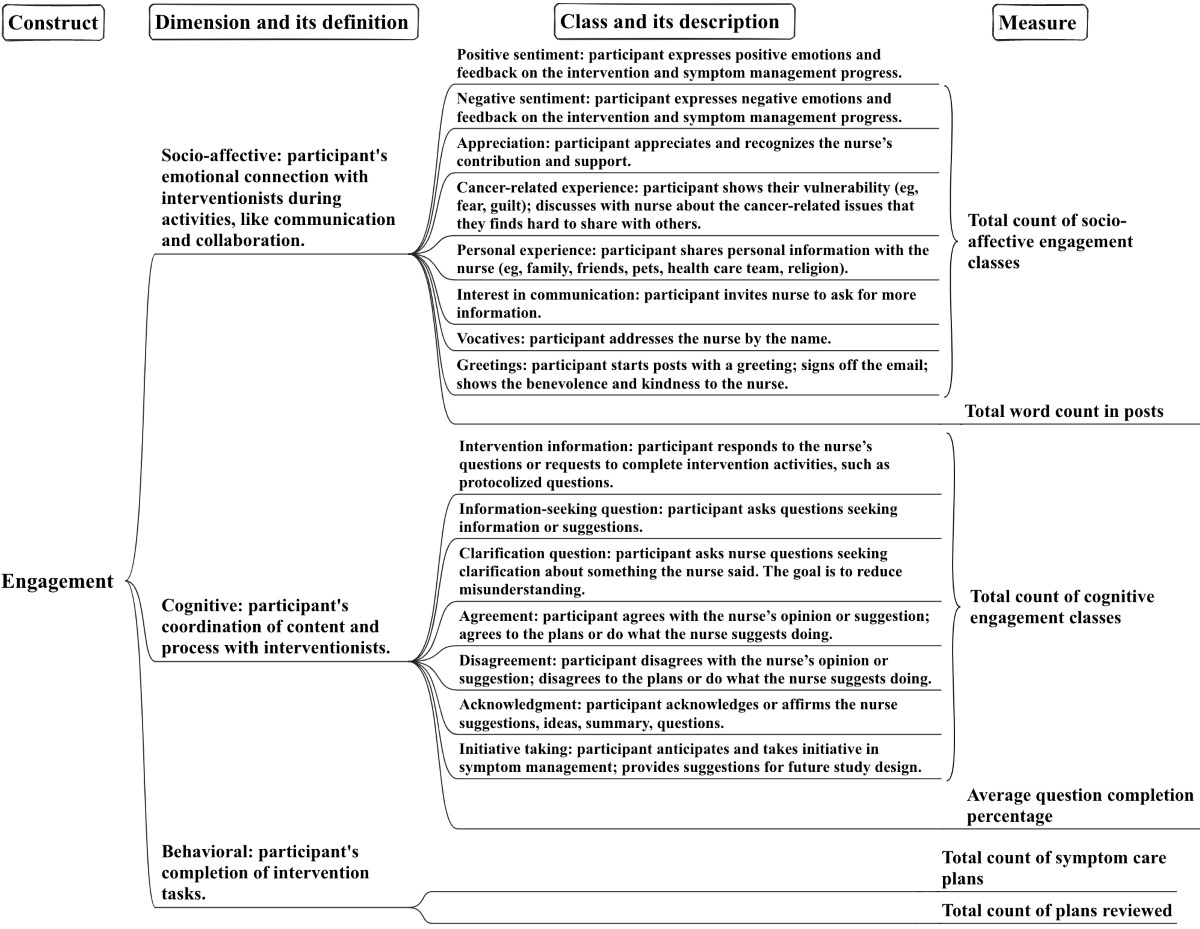
Conceptualizing and operationalizing engagement with provider-guided digital health interventions across socio-affective, cognitive, and behavioral dimensions.

In the previous study [28], we applied the SACe framework to identify linguistic signals and categorize engagement within patient-nurse communication on Nurse WRITE's asynchronous message boards. This analysis revealed 8 different socio-affective engagement classes (eg, expressing positive sentiment, sharing cancer-related experiences, showing interest in continued communication)—behaviors representing participants' efforts to build emotional connections with nurses. Additionally, we identified 7 cognitive engagement classes (eg, answering intervention questions, agreeing with the nurse’ suggestions, asking information-seeking questions)—behaviors that reflected participants' collaborative content and process coordination with nurses through written communication during the intervention. Figure 1 illustrates how engagement is conceptualized and operationalized in socio-affective, cognitive, and behavioral dimensions. The diagram progresses from left to right, beginning with conceptualizing engagement into 3 dimensions, each with its own accompanying definition. Subsequently, socio-affective engagement is operationalized into 8 distinct classes of behaviors; then, cognitive engagement is similarly operationalized into 7 classes, each with a corresponding description. The third dimension, behavioral engagement, is measured by 2 intervention milestones: symptom care plan creation and review. This process culminated in the development of 6 measures to assess engagement within these 3 dimensions quantitatively.

### Socio-Affective Dimension

In our prior study [28], we identified 8 classes of behaviors in the socio-affective dimension, representing participants' emotional connection efforts with the nurse. To quantify engagement in this dimension, we developed 2 measures. First, the total count of socio-affective engagement classes was computed by summing the number of instances of each class. Further descriptions of socio-affective engagement classes in participant posts can be found in Figure 1. In addition, the total word count served as a proxy for overall participant effort in communication and building connections with nurses [39], constituting the second socio-affective engagement measure.

### Cognitive Dimension

In our prior study [28], we also identified 7 classes in the cognitive dimension, demonstrating participants' efforts in coordinating intervention content and process with nurses. Engagement was quantified by summing the number of instances of each class, yielding the first cognitive measure: the total count of cognitive engagement classes. Full descriptions of each cognitive engagement class can be found in Figure 1. To obtain the second cognitive engagement measure, in this study, 2 trained raters independently analyzed the patients' posts to determine the question completion rate—reflecting cognitive engagement—by calculating the percentage of nurses' intervention-related questions and requests addressed by each participant on message boards. For instance, if a nurse poses 7 protocolized questions about symptom representation in her post (eg, “How severe is your symptom?” or “What have you tried to manage the symptoms?”), and the patient responds to 3 of these questions, their question completion rate would be 3 out of 7. This metric serves as a vital indicator of patient cognitive engagement, indicating their level of understanding and coordination of intervention activities with the nurse, regardless of participant verbosity. Inter-rater reliability of participant question completion percentage, assessed on 178 posts, yielded a Cohen κ score of 0.84.

### Behavioral Dimension

#### Overview

Website data was extracted to determine behavioral engagement measures: total count of symptom care plans and the total count of care plan reviews and revisions.

#### Aim 2: Apply the Framework to Categorize Patients' Engagement Patterns

We conducted a descriptive analysis of patient characteristics (eg, age and education). Following that, we quantified participants’ engagement with Nurse WRITE using the 6 measures across socio-affective, cognitive, and behavioral dimensions: total count of socio-affective engagement classes, total word count, total count of cognitive classes, average question completion percentage, total count of care plans, and plan reviews. Next, we used k-means clustering to categorize participants into distinctive groups based on these 6 measures. k-Means clustering is an unsupervised machine learning algorithm that aims to group data based on feature similarity, with the number of groups represented by K [40]. Additionally, we conducted a narrative analysis [41] of participants' posts to elucidate further engagement patterns within these distinctive groups, including understanding participants’ experiences and motivations.

## Results

### Sample Characteristics

The majority of participants included in this analysis were White (63/68; 93%), married or cohabitating (51/68; 75%), middle-aged (mean 59.7 (SD 9.5), range 24-83), and had a bachelor's degree (median 16, IQR 13-17, range 11-22 years of formal education). More than half of the participants were not working (37/68; 54%), including individuals who were disabled and unable to work, retired, and unemployed. On average, participants reported moderate symptom severity (5.3/10) and symptom distress (2.1/4). Over half of the sample (38/68; 56%) had at least one comorbidity.

### Aim 1: Finalize a Conceptual and Analytic Framework With Engagement Measures

On average, participants exhibited socio-affective engagement classes of behaviors, such as expressing positive sentiment and appreciation, 34.7 (SD 28.8) times. The participants also contributed substantially to the message boards, with an average word count of 14,851 (SD 14,064). Participants demonstrated dimensions of cognitive engagement behaviors such as answering protocolized questions, seeking information through inquiries, and agreeing with the nurse's opinions, on average, 19.4 times (SD 16.3). Furthermore, they maintained an average completion rate of 78.3% (SD 14.9%) when addressing nurses' questions or fulfilling intervention tasks. Regarding behavioral engagement, on average, participants completed 1.6 symptom care plans (SD 0.2) and 0.7 plan reviews (SD 0.9).

### Aim 2: Apply the Framework to Categorize Patients' Engagement Patterns

k-Means clustering analysis revealed the formation of 3 main groups characterized by their level of engagement in the 3 dimensions (ie, total count of socio-affective engagement classes, total word count, total count of cognitive engagement classes, question completion percentage, total count of symptom care plans, and care plan reviews completed). Given the nonnormal distribution of each engagement measure, we used the median and IQR to describe the engagement levels within each main group. Table 1 presents the median and IQR values for measures in the 3 engagement dimensions across each main group.

**Table 1:** Median and IQR of 6 engagement measures across 3 dimensions for high (n=13), moderate (n=17), and low engagers (n=38).

Engagement measures		High engagers	Moderate engagers	Low engagers
**Socio-affective dimension,** **median (IQR)**				
	Total count of socio-affective classes	81 (73-92)	39 (36-47)	13.5 (6.3-22.3)
	Total word count	36,956 (26,199-46,265)	18,034 (13,837-19,624)	6315.5 (2636.3-7358.3)
**Cognitive dimension,** **median (IQR)**				
	Total count of cognitive classes	46 (39-51)	25 (20-28)	7.5 (3.5-11.8)
	Average question completion percentage	91.7 (82.2-93.7)	86.4 (80-96.4)	68.3 (60.1-79.6)
**Behavioral dimension,** **median (IQR)**				
	Total count of symptom care plans	3 (3-3)	2 (2-3)	1 (0-1)
	Total count of plan reviews	2 (2-3)	1 (1-1)	0 (0-0)

There were 13 high engagers, 17 moderate engagers, and 38 low engagers. Regarding socio-affective engagement classes and the total word count, high engagers showed approximately 6 times higher engagement than low engagers and twice that of the moderate engagers. More specifically, high engagers established rapport by addressing nurses by name, expressing sincere appreciation, sharing personal experiences, displaying vulnerability, and expressing positive emotions towards the intervention, resulting in a high median total count of socio-affective engagement behaviors of 81 (IQR 73-92) times. In contrast, moderate (median 39, IQR 36-47) and low engagers (median 13.5, IQR 6.3-22.3) showed less interest in building personal connections with the nurse. Regarding the total word count, highly engaged participants wrote a median of 36,956 (IQR 26,199-46,265) words to their nurse, compared to moderate engagers (median 18,034, IQR 13,837-19,624) and low engagers (median 6315.5, IQR 2636.3-7358.3).

Cognitively, high engagers actively participated and demonstrated a strong commitment to learning symptom management techniques. They more consistently completed nurses' questions and requests (median 91.7%, IQR 82.2%-93.7%) compared to moderate engagers (median 86.4%, IQR 80%-96.4%) and low engagers (median 68.3%, IQR 60.1%-79.6%); sought information, acknowledged nurses' posts, agreed with suggestions, showing a median frequency of cognitive engagement of 46 (IQR 39-51), which is 6.1 times more often than low engagers and 1.8 times that of moderate engagers. Some high engagers even independently initiated second and third symptom care plans, sharing effective strategies and leading the intervention without prompts from the nurse. For example, one participant shared with the nurse the significance of prayer (which was not formally integrated as a strategy within the intervention), emphasizing its role in symptom management and coping with cancer. Compared to high engagers, the moderate engagers primarily focused on answering part of the intervention protocolized questions and agreeing with the nurse without gaining the confidence to lead themselves through the intervention.

Regarding behavioral engagement, high engagers completed a median of 3 (IQR 3-3) symptom care plans and 2 (IQR 2-3) revisions. Some of them exceeded intervention targets by working on the fourth or fifth symptoms. In contrast, moderate engagers completed a median of 2 (IQR 2-3) symptom care plans and 1 (IQR 1-1) review, whereas low engagers only completed 1 (IQR 0-1) symptom care plan and no reviews. Highly engaged participants, who formed an early rapport with nurses, often shared their insight and understanding following learning intervention materials, such as symptom care guides and common concerns for women with gynecological cancer. One patient wrote, “It was the first time I actually felt normal. When going to the doc, these symptoms have never even come up. I felt like I belonged! And really wasn't off my rocker.” This newfound perspective motivated her to actively pursue improved symptom management.

## Discussion

### Principal Results

Significant variations existed among engagers in socio-affective, cognitive, and behavioral dimensions, providing insights into how a provider-guided DHI operates. High engagers demonstrated approximately six-fold higher total word counts and SACe activities than low engagers and twice as much as moderate engagers. A positive feedback loop may exist between participant engagement, behavior change, and symptom management [1,42,43]. Enhanced emotional connections with nurses and increased written communication (socio-affective engagement) appear to contribute to a deeper understanding of intervention content and improved problem-solving skills (cognitive engagement). This synergy of SACe streamlines the development of highly personalized symptom management care plans (behavioral engagement) and the implementation of various strategies to adopt healthy behaviors and manage symptoms. Consequently, these factors motivate participants to engage further with the nurse and the intervention, as the literature suggests [42]. Prior reviews also underscored the significance of connecting engagement with a DHI to desired behavior change [1,21]. Using a combination of measures, we have taken the first steps in measuring this meaningful engagement within a provider-guided DHI. However, further research is warranted to investigate and validate the relationship between engagement across these 3 dimensions and patient symptom outcomes, including patient-perceived symptom control.

The observed variations in participants’ word counts may be related to the Nurse WRITE intervention's writing-intensive nature and differences in education levels among the groups. Limited computer literacy and busy work schedules may also have constrained participants’ ability and available time for message board contributions, aligning with prior research showing that education level, computer literacy, and employment can influence participants’ engagement with DHIs [42,44].

While significant differences in participants’ average question completion percentages were found among high, moderate, and low engagers, these differences were not as substantial as observed in other cognitive and socio-affective engagement measures. This is likely because some low and moderate engagers answered all the questions in their posts but logged on to the message board less frequently (and therefore had fewer posts), which artificially inflated their average completion percentage.

Regarding behavioral engagement, variations in the number of plans and plan reviews may be related to participants' perceptions of symptom severity and burden. Those who perceive more severe symptoms may be more likely to seek assistance from nurse interventionists to manage symptoms. In contrast, those who perceive milder symptoms may have prioritized work and family responsibilities or considered the intervention unnecessary. Further research is necessary to investigate and validate the impact of patient factors such as education, employment, and symptom perception on engagement levels to facilitate intervention tailoring.

Applying the DHI engagement framework to Nurse WRITE also provided valuable insights regarding the optimal duration and timing of provider-guided DHI for symptom management among highly disease-burdened individuals. Although the intervention aimed to address 3 target symptoms, 50% of high engagers could not complete the intervention goal (3 “target” care plan reviews), indicating a need for a more extended intervention period. Moderate and low engagers took longer to respond than high engagers, suggesting their potential to achieve the intervention goals with more time. These findings resonate with prior research on web-based distress management programs for cardiovascular patients, emphasizing the importance of investigating intervention duration and timing [45]. Our findings suggest that future digital symptom management interventions for advanced cancer patients should extend beyond 8 weeks to ensure maximum benefit.

### Limitations

This study adopted an exploratory, iterative approach to assessing patient engagement patterns owing to the limited and convenient sample size of 68 participants. Qualitative analysis unveiled subtleties in engagement patterns that extend beyond the 3 main groups identified by k-means clustering. Certain participants exhibited high levels of socio-affective engagement but demonstrated lower to moderate levels of cognitive engagement. Their emphasis leaned towards expressing emotions rather than active involvement with the intervention content aimed at care plan development, albeit such occurrences were infrequent. Additionally, the duration of Nurse WRITE was limited to 8 weeks, potentially insufficient for participants to fully achieve intervention goals. Future investigations with larger sample sizes and extended intervention periods are imperative to elucidate and build upon these findings comprehensively.

An alternative analysis of participants' communication and connection-building efforts could involve examining word count per post as a socio-affective engagement measure. However, the need for supporting linguistic literature for this approach presents a limitation. Furthermore, implementing such a measure may be challenging due to the diverse posting styles observed among Nurse WRITE participants. Some individuals favor numerous shorter posts, while others consolidate their responses into longer messages, potentially affecting the accuracy of the analysis.

This paper identifies and explains engagement patterns, proposing hypotheses regarding potential engagement influencers (eg, employment and symptom severity) for future investigation. Building upon these hypotheses, our future work will focus on elucidating group characteristics, examining engagement-influencing factors, and providing insights for tailoring interventions, such as adjusting intervention dosage.

### Comparison With Prior Work

Compared to traditional measures of cognitive and socio-affective engagement dimensions, such as exit interview interventions [26] or the number of postings [20], our engagement framework offers a more nuanced perspective. SACe activities provide real-time insights into participants' focus, interests, emotions, and actions on message boards, allowing us to quantify the intrinsic aspects of 2-way communication. These measures, along with the other socio-affective (total word count) and cognitive engagement measures (average question completion percentage), guide behavioral intervention scientists in assessing participants' efforts and the outcomes of 2-way interactions in a complex provider-guided DHI.

Expanding on this research direction, it's crucial to advance tools (eg, built upon MedNgage models [28]) to efficiently capture various engagement metrics in near real-time and provide suggestions for intervention tailoring. This advancement targets the reduction of labor-intensive retrospective qualitative analysis and addresses the methodological challenges outlined in the recent review [21]. One potential strategy is to incorporate a large language model (such as ChatGPT (OpenAI) or locally accessible alternatives) into the analysis of qualitative transcripts while taking measures to ensure patient data confidentiality. Such an approach could include summarizing patient-provider conversations and leveraging the outputs of MedNgage models [28] to produce detailed, easily comprehensible qualitative reports for nurse interventionists.

### Conclusions

Expanding upon prior research, we have developed a comprehensive framework for behavioral intervention scientists to analyze patient engagement in provider-guided DHIs. Using this framework on Nurse WRITE—a provider-guided DHI—we classified participants as high, moderate, and low engagers across the 3 dimensions of engagement (socio-affective, cognitive, and behavioral). This provides insights into the operational intricacies of a successful provider-guided DHI across various levels of participant engagement. Further research is essential to validate this framework with other provider-guided DHIs, explore the impact of patient factors (eg, education, employment, and symptom perception) on engagement, and assess how engagement influences the efficacy of the intervention. In the context of DHIs for symptom management among advanced cancer survivors, extending the intervention period beyond 8 weeks should be considered so that participants have more opportunities to engage and obtain the full benefits of the intervention.

## Abbreviations

DHI: digital health intervention
GOG: Gynecologic Oncology Group
SACe: socio-affective and cognitive engagement

## References

[1] Yardley L, Spring BJ, Riper H, Morrison LG, Crane DH, Curtis K, Merchant GC, Naughton F, Blandford A (2016). Understanding and promoting effective engagement with digital behavior change interventions. Am J Prev Med.

[2] Agboola S, Kamdar M, Flanagan C, Searl M, Traeger L, Kvedar J, Jethwani K (2014). Pain management in cancer patients using a mobile app: study design of a randomized controlled trial. JMIR Res Protoc.

[3] Parker S, Prince A, Thomas L, Song H, Milosevic D, Harris M, IMPACT Study Group (2018). Electronic, mobile and telehealth tools for vulnerable patients with chronic disease: a systematic review and realist synthesis. BMJ Open.

[4] Hong YA, Goldberg D, Ory MG, Towne SD, Forjuoh SN, Kellstedt D, Wang S (2015). Efficacy of a mobile-enabled web app (iCanFit) in promoting physical activity among older cancer survivors: a pilot study. JMIR Cancer.

[5] Sun L, Wang Y, Greene B, Xiao Q, Jiao C, Ji M, Wu Y (2017). Facilitators and barriers to using physical activity smartphone apps among Chinese patients with chronic diseases. BMC Med Inform Decis Mak.

[6] Wang Y, Sun L, Xu Y, Xiao Q, Chang P, Wu Y (2015). Comparision and analysis of top 10 exercise android Apps in mainland China. Stud Health Technol Inform.

[7] Demark-Wahnefried W, Schmitz KH, Alfano CH, Bail JR, Goodwin PJ, Thomson CA, Bradley DW, Courneya KS, Befort CA, Denlinger CS, Ligibel JA, Dietz WH, Stolley MR, Irwin ML, Bamman MM, Apovian CM, Pinto BM, Wolin KY, Ballard RM, Dannenberg AJ, Eakin EG, Longjohn MM, Raffa SD, Adams-Campbell LL, Buzaglo JS, Nass SJ, Massetti GM, Balogh EP, Kraft ES, Parekh AK, Sanghavi DM, Morris GS, Basen-Engquist K (2018). Weight management and physical activity throughout the cancer care continuum. CA Cancer J Clin.

[8] Viola A, Panigrahi G, Devine KA (2020). Digital interventions for adolescent and young adult cancer survivors. Curr Opin Support Palliat Care.

[9] Nahum-Shani I, Smith SN, Spring BJ, Collins LM, Witkiewitz K, Tewari A, Murphy SA (2018). Just-in-time adaptive interventions (JITAIs) in mobile health: key components and design principles for ongoing health behavior support. Ann Behav Med.

[10] Renfrew ME, Morton DP, Morton JK, Przybylko G (2021). The influence of human support on the effectiveness of digital mental health promotion interventions for the general population. Front Psychol.

[11] Kelders SM, van Zyl LE, Ludden GDS (2020). The concept and components of engagement in different domains applied to eHealth: a systematic scoping review. Front Psychol.

[12] Donkin L, Christensen H, Naismith SL, Neal B, Hickie IB, Glozier N (2011). A systematic review of the impact of adherence on the effectiveness of e-therapies. J Med Internet Res.

[13] Couper MP, Alexander GL, Zhang N, Little RJA, Maddy N, Nowak MA, McClure JB, Calvi JJ, Rolnick SJ, Stopponi MA, Cole Johnson C (2010). Engagement and retention: measuring breadth and depth of participant use of an online intervention. J Med Internet Res.

[14] Schubart JR, Stuckey HL, Ganeshamoorthy A, Sciamanna CN (2011). Chronic health conditions and internet behavioral interventions: a review of factors to enhance user engagement. Comput Inform Nurs.

[15] Arden-Close EJ, Smith E, Bradbury K, Morrison L, Dennison L, Michaelides D, Yardley L (2015). A visualization tool to analyse usage of web-based interventions: the example of positive online weight reduction (POWeR). JMIR Hum Factors.

[16] Chen Z, Koh PW, Ritter PL, Lorig K, Bantum EO, Saria S (2015). Dissecting an online intervention for cancer survivors: four exploratory analyses of internet engagement and its effects on health status and health behaviors. Health Educ Behav.

[17] Davies C, Duncan MJ, Vandelanotte C, Hall S, Corry K, Hooker C (2012). Exploring the feasibility of implementing a pedometer-based physical activity program in primary school settings: a case study of 10,000 steps. Health Promot J Austr.

[18] Morrison C, Doherty G (2014). Analyzing engagement in a web-based intervention platform through visualizing log-data. J Med Internet Res.

[19] Poirier J, Cobb NK (2012). Social influence as a driver of engagement in a web-based health intervention. J Med Internet Res.

[20] Han JY, Kim JH, Yoon HJ, Shim M, McTavish FM, Gustafson DH (2012). Social and psychological determinants of levels of engagement with an online breast cancer support group: posters, lurkers, and nonusers. J Health Commun.

[21] Short CE, DeSmet A, Woods C, Williams SL, Maher C, Middelweerd A, Müller AM, Wark PA, Vandelanotte C, Poppe L, Hingle MD, Crutzen R (2018). Measuring engagement in eHealth and mHealth behavior change interventions: viewpoint of methodologies. J Med Internet Res.

[22] Paz Castro R, Haug S, Filler A, Kowatsch T, Schaub MP (2017). Engagement within a mobile phone-based smoking cessation intervention for adolescents and its association with participant characteristics and outcomes. J Med Internet Res.

[23] Gouveia R, Karapanos E, Hassenzahl M (2015). How do we engage with activity trackers?: a longitudinal study of Habito.

[24] Lefebvre RC, Tada Y, Hilfiker SW, Cynthia B (2010). The assessment of user engagement with eHealth content: the eHealth engagement scale. J Comput Mediat Commun.

[25] Potts C, Bond R, Ryan A, Mulvenna M, McCauley C, Laird E, Goode D (2020). Ecological momentary assessment within a digital health intervention for reminiscence in persons with dementia and caregivers: user engagement study. JMIR Mhealth Uhealth.

[26] Crafoord M, Fjell M, Sundberg K, Nilsson M, Langius-Eklöf A (2020). Engagement in an interactive app for symptom self-management during treatment in patients with breast or prostate cancer: mixed methods study. J Med Internet Res.

[27] Martey R, Kenski K, Folkestad J, Feldman L, Gordis E, Shaw A, Stromer-Galley J, Clegg B, Zhang H, Kaufman N, Rabkin An, Shaikh S, Strzalkowski T (2014). Measuring game engagement. Simul Gaming.

[28] Wang Y, Donovan H, Hassan S, Alikhani M (2023). MedNgage: a dataset for understanding engagement in patient-nurse conversations. https://aclanthology.org/2023.findings-acl.282/.

[29] Donovan HS, Sereika SM, Wenzel LB, Edwards RP, Knapp JE, Hughes SH, Roberge MC, Thomas TH, Klein SJ, Spring MB, Nolte S, Landrum LM, Casey AC, Mutch DG, DeBernardo RL, Muller CY, Sullivan SA, Ward SE (2022). Effects of the write symptoms interventions on symptoms and quality of life among patients with recurrent ovarian cancers: an NRG oncology/GOG study (GOG-0259). J Clin Oncol.

[30] Donovan HS, Ward SE, Song M, Heidrich SM, Gunnarsdottir S, Phillips CM (2007). An update on the representational approach to patient education. J Nurs Scholarsh.

[31] Donovan HS, Ward S, Sherwood P, Serlin RC (2008). Evaluation of the symptom representation questionnaire (SRQ) for assessing cancer-related symptoms. J Pain Symptom Manage.

[32] Swan K, Garrison (2009). A constructivist approach to online learning: the community of inquiry framework. Information Technology and Constructivism in Higher Education: Progressive Learning Frameworks.

[33] Clark H, Brennan S. (1991). Grounding in communication. Perspectives on socially shared cognition.

[34] Asher N, Asher N (2003). Logics of Conversation. Studies in Natural Language Processing.

[35] Oken MM, Creech RH, Tormey DC, Horton J, Davis TE, McFadden ET, Carbone PP (1982). Toxicity and response criteria of the eastern cooperative oncology group. Am J Clin Oncol.

[36] Rubin S (2004). Chemotherapy of Gynecologic Cancers. Society of Gynecologic Oncologists Handbook, 2nd edition.

[37] Dumrongpakapakorn P (2011). The development of a comprehensive fidelity monitoring plan for a web-based psycho-educational intervention: The WRITE symptoms study. Doctoral dissertation.

[38] Sereika S, Engberg S (2016). Development of Standardized Sociodemographic and Co-Morbidity Questionnaires.

[39] Tausczik Y, Pennebaker Jw (2009). The psychological meaning of words: LIWC and computerized text analysis methods. J Lang Soc Psychol.

[40] Pedregosa F, Varoquaux G, Gramfort A, Michel V, Thirion B, Grisel O, Blondel M, Prettenhofer P, Weiss R, Dubourg V, Vanderplas J, Passos A, Cournapeau D, Brucher M, Perrot M, Duchesnay M (2011). Scikit-learn: machine learning in Python. J Mach Learn Res.

[41] Stephens C, Breheny M (2012). Narrative analysis in psychological research: an integrated approach to interpreting stories. Qual Res Psychol.

[42] Perski O, Blandford A, West R, Michie S (2017). Conceptualising engagement with digital behaviour change interventions: a systematic review using principles from critical interpretive synthesis. Transl Behav Med.

[43] Peeler A, Nelson K, Agrawalla V, Badawi S, Moore R, Li D, Street L, Hager DN, Dennison Himmelfarb C, Davidson PM, Koirala B (2024). Living with multimorbidity: a qualitative exploration of shared experiences of patients, family caregivers, and healthcare professionals in managing symptoms in the United States. J Adv Nurs.

[44] Vandelanotte C, Müller AM, Short CE, Hingle M, Nathan N, Williams SL, Lopez ML, Parekh S, Maher CA (2016). Past, present, and future of eHealth and mHealth research to improve physical activity and dietary behaviors. J Nutr Educ Behav.

[45] Habibović M, Cuijpers P, Alings M, van der Voort P, Theuns D, Bouwels L, Herrman JP, Valk S, Pedersen S (2014). Attrition and adherence in a WEB-based distress management program for implantable cardioverter defibrillator patients (WEBCARE): randomized controlled trial. J Med Internet Res.

